# Emergence of Young Human Genes after a Burst of Retroposition in Primates

**DOI:** 10.1371/journal.pbio.0030357

**Published:** 2005-10-11

**Authors:** Ana Claudia Marques, Isabelle Dupanloup, Nicolas Vinckenbosch, Alexandre Reymond, Henrik Kaessmann

**Affiliations:** **1**Center for Integrative Genomics, University of Lausanne, Lausanne, Switzerland,; **2**Department of Genetic Medicine and Development, University of Geneva Medical School, Geneva, Switzerland; University of DublinIreland

## Abstract

The origin of new genes through gene duplication is fundamental to the evolution of lineage- or species-specific phenotypic traits. In this report, we estimate the number of functional retrogenes on the lineage leading to humans generated by the high rate of retroposition (retroduplication) in primates. Extensive comparative sequencing and expression studies coupled with evolutionary analyses and simulations suggest that a significant proportion of recent retrocopies represent bona fide human genes. We estimate that at least one new retrogene per million years emerged on the human lineage during the past ∼63 million years of primate evolution. Detailed analysis of a subset of the data shows that the majority of retrogenes are specifically expressed in testis, whereas their parental genes show broad expression patterns. Consistently, most retrogenes evolved functional roles in spermatogenesis. Proteins encoded by X chromosome−derived retrogenes were strongly preserved by purifying selection following the duplication event, supporting the view that they may act as functional autosomal substitutes during X-inactivation of late spermatogenesis genes. Also, some retrogenes acquired a new or more adapted function driven by positive selection. We conclude that retroduplication significantly contributed to the formation of recent human genes and that most new retrogenes were progressively recruited during primate evolution by natural and/or sexual selection to enhance male germline function.

## Abstract 2

In humans, retroposition--integration into the genome of DNA reverse transcribed from mRNA--has contributed to the formation of recent functional genes selected to enhance male germline function.

## Introduction

Together with more subtle genetic modifications such as gene expression changes and point substitutions, new genes with novel functions may have significantly contributed to the evolution of new phenotypes specific to humans and their closest evolutionary relatives. New duplicate genes may originate through (segmental) gene duplication by intra- or interchromosomal transposition of gene-containing segments [1,2]. Another mechanism, retroposition, generates new intronless gene copies (retrocopies) by reverse transcription of mRNAs derived from source genes (“parental” genes), followed by reintegration of the resulting cDNA in the genome [2,3]. Retroposition was commonly thought to generate nonfunctional gene copies (retropseudogenes) that accumulate disablements such as premature stop codons and frameshift mutations [4], because the copied mRNA is generally lacking regulatory elements. However, we and others have recently shown that retroposition has generated a significant number of new functional genes (retrogenes) in mammalian and invertebrate animal genomes [3,5,6].

Multiple studies have suggested a high rate of retroposition on the primate and rodent lineages [7−9], probably driven by the activity of L1 retrotransposable elements [10]. Thus, retroposition may also have provided abundant raw material for the formation of new genes on the primate lineage leading to humans, potentially generating many more retrogenes than the four primate-specific retrogenes (present in the human genome) with functional roles and/or expression in testis, brain, and lymphocytes previously described [11−14].

To assess the importance of retroposition for the creation of new genes on the primate lineage leading to humans, we systematically screened the human genome for retrogenes that emerged during the primate burst of retroposition. Our results suggest an important role of retroposition in the formation of new genes and phenotypes in the recent evolution of the human genome.

## Results

### Age Distribution of Human Retrocopies

**Figure 1:**
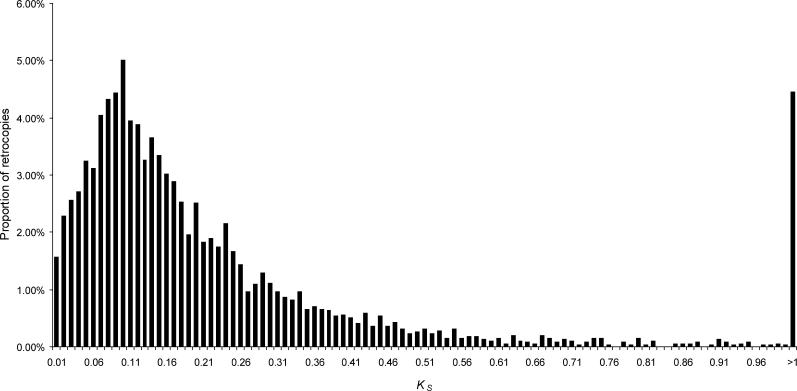
K_S_ Distribution for 3,951 Retrocopies The peak at *K*_S_ ≍ 0.1 suggests a burst of retroposition on the primate lineage (see also text and Figure S1). Retrocopies with *K*_S_ > 1 were pooled in a single bin.

We identified 3,951 retrocopies (and their corresponding parental genes) in the human genome using a refinement of a previously published procedure [5] (see Materials and Methods). Among these, 705 retrocopies (∼18%) are found to be “intact,” i.e., they show no disablements such as premature stop codons or frameshift mutations when compared to the open reading frame (ORF) of their parental genes. To assess the age distribution of retrocopies, we calculated nucleotide divergence at silent sites (*K*_S_) between retrocopies and their parental genes (Figure 1). Assuming neutral mutation rates of 1−1.3 × 10^−9^ substitutions per site per year [15], the high number of retrocopies with *K*_S_ ≍ 0.1 suggests that the burst of retroposition reached its peak approximately 38−50 million years ago (MYA) on the primate lineage, in agreement with previous estimates [7,8]. The vast majority of retrocopies (91%) also show a divergence at silent sites much lower than that observed between human and mouse genes (Figures 1 and S1), indicating that they arose after the human−mouse split. Therefore, our data are consistent with a high retroposition activity on the primate lineage.

### Rate of New Retrogene Formation

To estimate the number of recent human retrogenes, we compared signatures of selective constraint between intact, potentially functional retrocopies, and retropseudogenes (assumed to be nonfunctional, i.e., evolving neutrally). To this end, we calculated the ratio of nonsynonymous to synonymous substitutions per site (*K*_A_/*K*_S_) for retrocopy/parental gene pairs with a synonymous divergence of less than 0.15. This value approximately reflects the deepest neutral divergence in the primate tree between humans and the most divergent extant primate lineage [16,17], the lemurs, and corresponds to around 63 million years of primate evolution [18].

**Figure 2:**
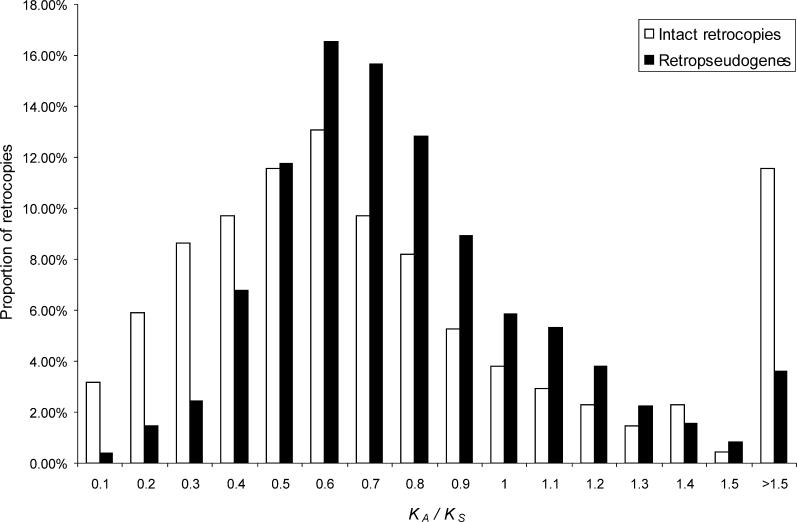
K_A_ */K*_S_ Distributions for 475 Intact Retrocopies and 1,554 Retropseudogenes with *K*_S_ < 0.15 Note that we tested that the differences observed for *K*_A_ */K*_S_ < 0.5 are not explained by differences in GC content (see Materials and Methods for details). The bin with *K*_A_ */K*_S_ > 1.5 includes estimates where *K*_S_ = 0 (*K*_A_ */K*_S_ = ∞).

This analysis reveals a difference in the *K*_A_/*K*_S_ distributions between intact copies and retropseudogenes (which may show low *K*_A_/*K*_S_ ratios by chance), with a highly significant excess of intact copies for *K*_A_/*K*_S_ < 0.5 (Figure 2; *p* < 10^−6^, Fisher's exact test). *K*_A_/*K*_S_ significantly less than one is indicative of purifying selection [19]. However, in a pairwise analysis, where *K*_A_/*K*_S_ reflects the average selective constraint on the retrocopy and parental gene, *K*_A_/*K*_S_ < 0.5 is indicative of purifying selection (i.e., *K*_A_/*K*_S_ < 1) on both copies [5]. The 16% excess of intact retrocopies relative to retropseudogenes at *K*_A_/*K*_S_ < 0.5 corresponds to approximately 76 retrogenes that were fixed on the primate lineage leading to humans through natural selection in the past 63 million years.

Based on a subset of the data for which a mouse ortholog of the human parental gene is available as an outgroup, we performed a similar analysis to calculate the *K*_A_/*K*_S_ ratio on the retrocopy lineage itself (Figure S2). Again, we observed a significant excess (*p* < 2 × 10^−3^) of intact retrocopies with low *K*_A_/*K*_S_ values. When we extrapolate this excess to the whole dataset (475 intact retrocopies with *K*_S_ < 0.15), this indicates that approximately 57 retrogenes in the human genome emerged in primates. This result is similar to the estimate based on the whole dataset using the pairwise *K*_A_/*K*_S_ approach.

Together, these analyses suggest that approximately one retrogene per million years has emerged on the primate lineage leading to humans. It should be noted that the estimates based on this approach are restricted to cases with low *K*_A_/*K*_S_ values averaged over the entire sequence, despite the fact that retrogenes may be found with higher *K*_A_/*K*_S_ values due to the action of positive selection, a neutral phase of evolution upon emergence, or both (see Discussion).

### Functional Retrogenes

To identify and characterize individual functional retrogenes in the human genome that emerged recently in primate evolution, we selected 38 intact retrocopies with low divergence at silent sites from their parental genes (*K*_S_ < 0.15) for further study (Table S1). To obtain an unbiased view of new retrogene formation, we chose these retrocopies independent of their average pairwise *K*_A_/*K*_S_ values, as new genes may show high, intermediate, or low *K*_A_/*K*_S_ values, depending on the type and extent of selection acting after the duplication event [3]. We determined the age of the 38 retrocopies by screening for their presence or absence in eight primate genomes.

This phylogenetic dating approach revealed retrocopies that emerged throughout primate evolutionary history (Table S1). For instance, we identified five retrocopies present in all Old World primates, five hominoid-specific retrocopies, and six copies unique to humans. Our dating revealed that the *PGAM3* retrogene, previously shown to have been shaped by positive selection [11], originated recently in the ancestor of humans and the African apes, less than 14 MYA [18]. We also found that the *PABP3* retrogene, for which a function in testis was recently demonstrated [20], emerged in primates.

In order to identify functional retrogenes among these dated retrocopies, we used an approach that combines comparative genomic sequencing and evolutionary simulations. First, we selected only retrocopies with a minimum sequence length (>600 bp) and age (>8 million years; i.e., presence in humans and African apes), characteristics estimated to provide sufficient statistical power for the simulation approach (see Materials and Methods). We sequenced these copies in all species carrying them. Sequence alignments show that eight of these 23 retrocopies are intact in all species, whereas the remaining copies carry one or more stop codons and/or frameshift mutations in one or more lineages (Table 1).

**Table 1:**
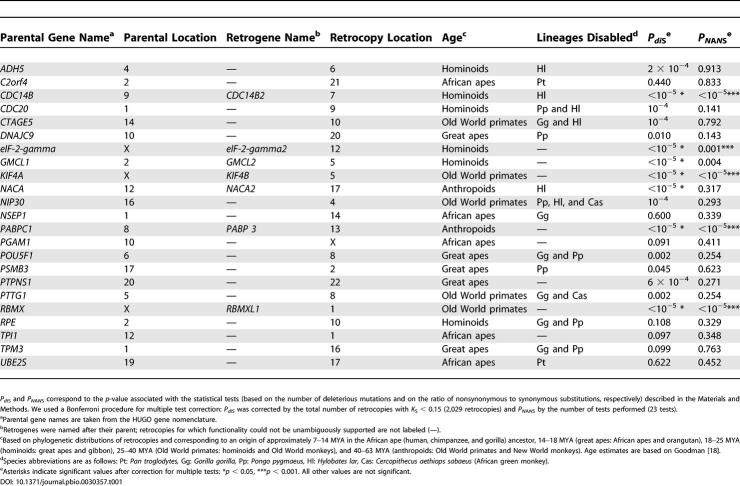
Retrocopies Tested for Identifying Retrogenes

Next, we used a simulation approach (see Materials and Methods), which is based on the basic assumption that under neutrality, an intact retrocopy will accumulate deleterious mutations (stop codons or frameshifts) over time that will disrupt its ORF and may eventually preclude gene function, whereas under functional constraint, natural selection will prevent the accumulation of deleterious mutations in the retrocopy sequence.

Our simulation approach estimates the probability that a gene copy would have retained its ORF since the duplication event, in all or most species in which it is present, if it had evolved neutrally along all lineages of the species tree. In parallel, this approach tests whether the number of nonsynonymous substitutions that accumulated since the retroposition event along the different branches of the species phylogeny is consistent with neutral evolution.

**Figure 3:**
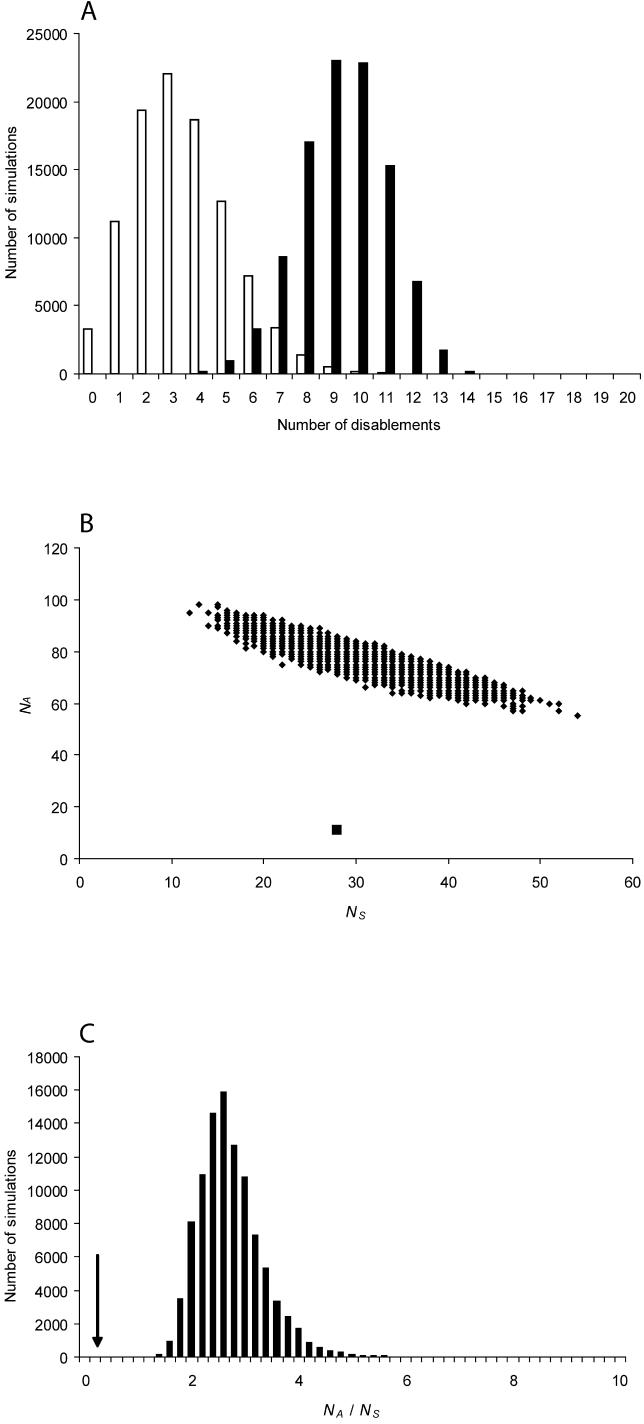
Illustration of the Simulation Results Used to Support Functionality of Retrogenes for One Case *(RBMXL1)* (A) Distribution of the number of disablements observed in 10^5^ simulations of the *RBMXL1* retrogene evolution under neutrality. The frequency distribution of stop codons is shown in white, and that of deleterious indels in black. All of the simulations showed at least one mutation disrupting the ORF (see text); simulations without stop codons all showed several frame-disrupting indels (the minimum number of such indels in each simulation is four). (B) Nonsynonymous (*N*_A_) and synonymous (*N*_S_) substitutions observed in 10^5^ simulations of neutral *RBMXL1* retrogene evolution (diamonds). The black square indicates the observed nonsynonymous and synonymous substitutions in the *RBMXL1* primate phylogeny. (C) Ratio of nonsynonymous to synonymous substitutions in 10^5^ simulations of *RBMXL1* retrogene evolution in the primate lineages. The arrow indicates the observed ratio of nonsynonymous to synonymous substitutions in the *RBMXL1* primate phylogeny.

The simulations revealed that seven retrocopies are unlikely to have remained intact in all (or most) species if they had evolved neutrally throughout their evolutionary history, even after correcting for multiple tests (*p* < 0.05; Table 1; Figures S3−S8). For example, a retrocopy on Chromosome 1 *(RBMXL1),* which we find to be intact in all six Old World primates carrying it, showed at least one disablement in each of 10^5^ simulations during which the retrocopy was evolving neutrally after the duplication event (Figure 3A). This strongly suggests that the ORFs of all seven retrocopies were selectively preserved after duplication. Therefore, these copies very likely represent functional genes. Among these seven genes is also *PABP3,* for which a functional protein has been previously described [20], confirming that our simulation approach correctly predicts the functionality of recent genes.

Five of the seven copies accumulated fewer nonsynonymous substitutions than expected under neutrality, lending further support to the notion that these copies were preserved through natural selection (Figures 3B, 3C, and S3−S8). The remaining genes *(NACA2* and *GMCL2)* may have been affected by positive selection at a subset of sites or may have experienced a period of relaxed selective constraint after duplication, rendering the average number of nonsynonymous substitutions not significantly different from that expected under a neutral evolutionary process.

The seven retrogenes identified here (Table 1) originated between 18 and 63 MYA [18] in the ancestor of hominoids *(CDC14B2, eIF-2-gamma2,* and *GMCL2),* Old World primates *(RBMXL1* and *KIF4b),* and anthropoid primates *(NACA2* and *PABP3).* On the basis of the functions of their parental genes (Table S1) or gene family members [20−28], these retrogenes can be predicted to play diverse functional roles in RNA processing and transport *(RBMXL1),* initiation of translation *(eIF-2-gamma2* and *PABP3),* mRNA stability *(PABP3),* transcriptional regulation and protein biosynthesis (*CDC14B2, GMCL2,* and *NACA2),* and chromosome condensation and segregation *(KIF4b).*

### Evolutionary Fate of New Retrogenes

Newly emerged retrogenes may evolve new functional roles through adaptive evolution of encoded proteins and/or by developing new spatial or temporal expression patterns. To trace the functional adaptation of the seven novel retrogenes identified here, we reconstructed phylogenetic trees based on the primate retrocopy and parental gene sequences and then scrutinized substitutional patterns on the retrogene branches in a maximum likelihood selection framework (Table 2). We also analyzed spatial gene expression patterns in 20 human tissues using RT-PCR.

**Table 2:**
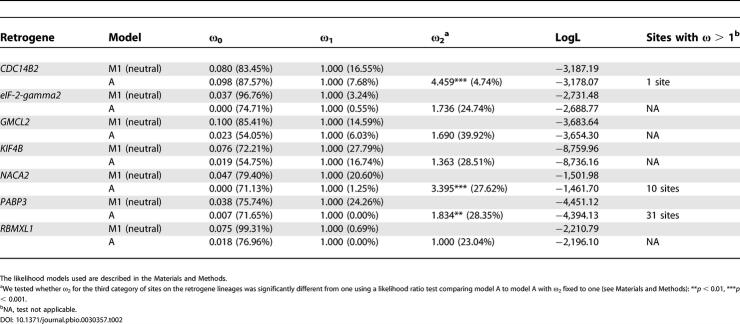
PAML Analyses for the Seven Retrogenes Identified by the Simulations Approach

**Figure 4:**
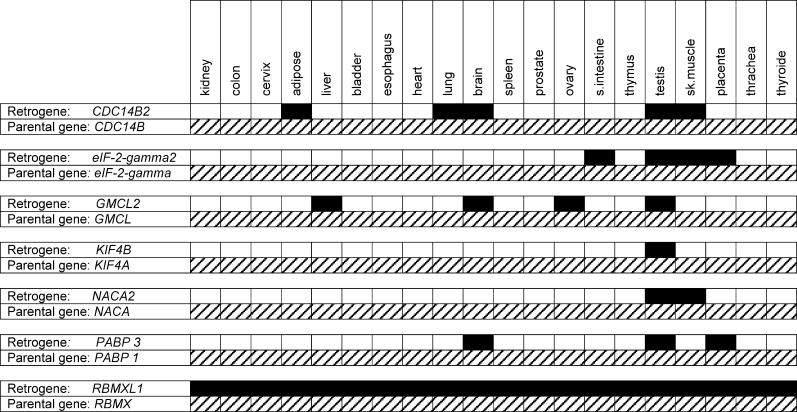
Expression Pattern of Retrogenes and Parents Determined by RT-PCR Black boxes indicate retrogenes; hatched boxes indicate parental genes. Note that in all cases testis expression of the retrogene was the strongest, as indicated by the semiquantitative PCR procedure (data not shown).

Strikingly, we found that all seven retrogenes are exclusively or predominantly transcribed in testis, whereas transcripts of their parental genes were detected in all tissues tested (Figure 4). Three of these retrogenes *(eIF-2-gamma2, RBMXL1,* and *KIF4b)* derive from parental genes located on the X chromosome (see Table 1). Our selection analyses show that substitutional models allowing for sites under purifying selection and neutrally evolving sites on the retrogene lineages after the duplication event provide the best fit for these genes. In agreement with our simulations (Table 1), purifying selection has shaped most of their codons (54%−77%; see Table 2), which suggests that ancestral/parental protein functions are likely preserved in these genes.

We have previously shown that X chromosomal genes in mammals generated a statistically significant excess of (autosomal) retrogenes relative to genes on other chromosomes [5]. One possible explanation for this pattern was that X chromosomal genes produced functional counterparts on autosomes that can be recruited during male meiosis when X chromosomal genes are silenced or during haploid stages of spermatogenesis [29,30]. Our findings that the coding sequences of the three recent X-derived genes identified here appear to be preserved by purifying selection at early stages of their evolution and that all genes are expressed (exclusively or most strongly) in testis (Figure 4) lend further support to this hypothesis. These retrogenes *(eIF-2-gamma2, RBMXL1,* and *KIF4b)* also support our previous notion that the generation of functional autosomal substitutes for genes on the X chromosome is an ongoing process [5]. In fact, this gene “movement” appears to have progressively enhanced male germline functions in primate evolution.

The four remaining genes stem from autosomes (see Table 1). Interestingly, the *Drosophila* ortholog *(germ cell-less)* of *GMCL1*—the parental gene of the hominoid-specific retrogene *GMCL2* identified here—was shown to be essential for germ cell formation [26,31,32]. Furthermore, the mouse ortholog of *GMCL1* [33] shows its highest expression in testis and has been shown to function as a transcriptional repressor [27]. Together, these results suggest that *GMCL2* might have been preserved through male selection to enhance testis function in hominoids.

The other three retrogenes *(CDC14B2, NACA2,* and *PABP3)* show a statistically significant excess of nonsynonymous to synonymous substitutions (*K*_A_/*K*_S_ > 1, *p* < 0.01) for a subset of sites (∼4.7%, ∼27.6%, and ∼28.4% of sites, respectively), indicative of accelerated protein evolution driven by positive Darwinian selection (see Table 2). This may suggest new or more adapted functional roles of these retrogenes in transcriptional regulation and protein biosynthesis in testis.

**Figure 5:**
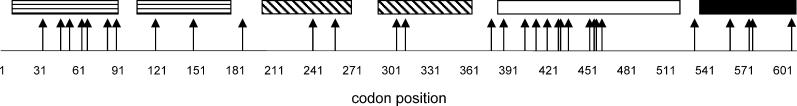
Evidence for Positive Selection on *PABP3* Codons The rectangles correspond to the different domains of the PABP1 protein: RNA poly(A)-tail-binding domains (vertical lines), RNA-binding domains (hatched), protein–protein interaction domain (white), and PABP homo-oligomerization domain (black). Positions of positively selected codons with high posterior probabilities (>0.95) are indicated by arrows.

For *PABP3,* the maximum likelihood procedure identifies many codons as being positively selected (Table 2; Figure 5). Positively selected sites are present in all major domains of the *PABP3*-encoded protein such as the poly(A)-binding domain (Figure 5). Interestingly, a recent study not only supports the presence and functionality of the *PABP3-*encoded protein but also provides evidence for altered poly(A)-binding affinity [20]. However, positively selected sites particularly cluster in a region that was shown in PABP proteins to be involved in interactions with not only other proteins such as translation initiation factors but also viruses that target this region to shut off protein synthesis in the host cell (Figure 5) [20]. This may indicate that PABP3 has evolved new or enhanced protein interaction properties and/or an altered viral susceptibility compared to its parent, PABP1. Testis expression of *PABP3* appears to be restricted to a later phase of spermatogenesis, during which the activity of *PABP1* is repressed [20]. This suggests that *PABP3* functionally replaces its parent to enhance translation and/or RNA stability during male meiosis.

*PABP3* provides an intriguing example of a retrogene that has adapted functionally by evolving a new spatial and temporal expression pattern as well as new protein properties relative to its parent. We have shown that this adaptation was driven by positive selection and occurred within the past ∼35−63 million years since the duplication event that gave rise to this gene in the common ancestor of anthropoid primates [18]. The high *K*_A_/*K*_S_ ratio (2.8) on the human lineage after the separation from that of the chimpanzee (Figure S9) might suggest that adaptation shaped human *PABP3* properties until recently in human evolution.

## Discussion

### Functional Retrogenes

Although gene duplications of different types have been prevalent in primate evolution, a more detailed picture with respect to the functionality of individual gene copies and their potential to contribute to human- and/or primate-specific phenotypes is only beginning to emerge [12,13,34−38]. Demonstrating the functionality of recently duplicated genes is hampered by their close similarity to original copies, which complicates both statistical and experimental inferences. Here, we have used a combination of comparative genomic sequencing, evolutionary analysis, and gene expression experiments to estimate the number of recent human genes that arose by retroposition and to characterize their functions.

Our study almost triples the number of described primate-specific retrogenes from four to 11 [11−14]. However, on the basis of a systematic analysis of selective signatures in retrocopy sequences, we estimate that approximately 57−76 retrogenes emerged during and after the primate burst of retroposition. This tentative estimate represents a lower bound for several reasons. First, our in silico approach (comparing *K*_A_/*K*_S_ values between intact and retropseudogene copies) only detects copies with low *K*_A_/*K*_S_ values, whereas newly emerged genes often show higher *K*_A_/*K*_S_ values owing to the action of positive selection at a subset of sites (*K*_A_/*K*_S_ > 1) and/or a neutral phase of evolution after duplication [3,12]. Second, retrocopies with disablements in their ORFs (as defined by their parents) are treated as pseudogenes in this analysis, although new retrogenes may emerge from truncated coding regions [3,13]. It is also known that new splicing signals in a coding region that contains frameshifts or premature stop codons may evolve to define a new intron or to generate chimeric transcripts with nearby or “host” genes [3]. Finally, duplicate “pseudogene” copies may play functional roles by virtue of their RNAs regulating closely related paralogous genes [39,40]. At any rate, our results suggest that in addition to other types of duplications [1], retroposition significantly contributed to new gene formation in primates.

### Retrogenes and Male Functions

It is remarkable that all seven retrogenes identified in this report are expressed predominantly or exclusively in testis, whereas their parents are all expressed ubiquitously. A preliminary survey of retrocopy transcription using expressed sequence tag databases suggests that this observation may reflect a general pattern (data not shown). Several factors may contribute to this effect. For example, chromatin remodeling [41] and abundance of RNA polymerase II complexes during late phases of male meiosis [42] lead to a state of “hypertranscription” [43], which may allow retrocopies to become initially transcribed in testis. This may also have facilitated transcription of new genes arising from pericentromeric segmental duplications [44,45]. Thus, there is a mechanistic bias that may favor testis expression of new genes.

However, our results suggest that testis expression is often not merely a by-product of new retrogene formation but that natural selection may have favored the recruitment of testis-specific regulatory elements to enhance the beneficial effects of the initial mechanistically driven testis transcription. Consistently, we can infer a testis function for five of the seven primate retrogenes identified here and for two of the four previously identified retrogenes (*TAF1L* and *UTP14C;* [13,14]). Five retrogenes *(eIF-2-gamma2, RBMXL1, KIF4b, TAF1L,* and *UTP14C)* stem from the X chromosome and probably either substitute for their parental genes during male meiosis [30] or otherwise enhance male germline function [46]. For one retrogene *(GMCL2),* a function in sperm formation can be postulated based on studies of parental orthologs. Finally, *PABP3* functionally adapted to late spermatogenesis both on the protein sequence level and by developing a highly specific expression pattern [20].

Sex- and reproduction-related genes are generally recognized as a class of rapidly evolving genes, particularly genes involved in male reproduction [47]. Possible causes include sperm competition, sexual conflict, and selection for reproductive isolation [48]. A comparison of the human and mouse genomes revealed an excess of lineage-specific expansions of genes related to reproduction as well as an accelerated protein evolution of such genes [49]. Together, these observations suggest that duplicate gene copies may have provided important raw material for rapid testis evolution in primates. Specifically, gene duplication may allow one copy of the duplicate pair to specialize in testis function, while the other is selectively preserved to sustain a role in somatic tissues [50−52]. Our data suggest that retroduplication may have provided a means to allow for such decoupling of functions in primates. Indeed, we show that selection to attain enhanced male germline function has progressively fixed and adapted retroposed gene copies on the primate lineage leading to humans.

## Materials and Methods

### Retrocopy screen

We retrieved all peptide sequences (categories: known and novel) from the Ensembl ([53]; http://www.ensembl.org/index.html) database (version 29). To screen for retrocopies, these peptide sequences were used as queries in translated similarity searches against the complete human genome (NCBI genome release 35) sequence using tBLASTn [54]. Adjacent homology matches were merged in a series of parsing steps using Perl scripts, combining only nearby matches (distance < 40 bp) that were likely not separated by introns. We also required that query and merged target sequences had significant similarity on the amino acid level (amino acid identity > 50%) and aligned to one another over more than 70% of the length of their sequence (minimum length: 50 amino acids). Next, we performed similarity searches of the merged sequences against all Ensembl genes (intron-containing and intronless) using FASTA. We kept only copies where the closest hit was an Ensembl peptide with multiple coding exons (putative parental gene). Merged sequences for which the closest match was an intronless gene were excluded from the data (e.g., to avoid intronless genes of other types such as olfactory receptor genes). We also confirmed the absence of introns in these retrocopies by mapping parental intron locations onto the alignments. We required that parental introns map within the alignments between parents and retrocopies and be larger than 80 bp. This threshold was chosen to ensure that real introns are missing in the retrocopies; 80 bp is larger than the gap size (40 bp) allowed in the merging step, it avoids mapping of small gaps in parental exons erroneously annotated as introns, and it takes into account that the majority of human introns are ∼80 bp or larger [55].

### Samples

Primate DNA samples were mainly obtained from the E CACC repository (Wiltshire, United Kingdom): chimpanzee *(Pan troglodytes),* gorilla *(Gorilla gorilla),* orangutan *(Pongo pygmaeus),* gibbon *(Hylobates lar),* Old World monkey (African green monkey, *Cercopithecus aethiops sabaeus*), and New World monkey (owl monkey, *Aotus trivirgatus*). Lemur *(Lemur catta)* and tupaia *(Tupaia glis)* DNA samples were obtained from Institut des Sciences de l'Evolution, Montpellier University 2.

### PCR and sequencing reactions

PCR amplifications were performed in a Mastercycler gradient (Eppendorf, Hamburg, Germany) using either *Taq* DNA Polymerase or ProofStart DNA Polymerase from Qiagen (Valencia, California, United States). PCRs were performed according to the instructions of the manufacturer. For sequencing, amplified PCR products were reamplified using a pair of nested primers. The resulting PCR products were purified using the MinElute PCR Purification Kit or QIAquick Gel Extraction Kit from Qiagen. From these PCR products, both strands of the retrogene coding sequence were determined using the BigDye 3.1 cycle sequencing kit (PerkinElmer, Wellesley, California, United States). The sequencing reactions were run on an ABI 3730 automated sequencer (Applied Biosystems, Foster City, California, United States). Parental and retrogene expression patterns were analyzed using PCR and a cDNA panel of 20 different human tissues. Experiments were repeated twice to confirm the expression pattern. Unique primer pairs were designed for both parental gene and retrogene, based on ClustalX alignments of parental and retrogene cDNA sequences. The cDNA panel was synthesized using the FirstChoice Human Total RNA Survey panel from Ambion (Austin, Texas, United States) and a SuperScript II First-Strand Synthesis System RT-PCR (Invitrogen, Carlsbad, California, United States). Reactions without reverse transcriptase were done in parallel as negative controls for all 20 tissues. RT-PCR amplifications were performed in a Mastercycler gradient (Eppendorf) using JumpsStart DNA Polymerase (Sigma-Aldrich, St. Louis, Missouri, United States) using standard conditions as recommended by the supplier. Products were purified using the MinElute PCR Purification Kit from Qiagen and sequenced using the same pair of primers. Obtained sequences for each retrogene were then aligned with both retrogene and parental gene sequences using ClustalX. To ensure that RT-PCR products were derived from the retrogene, nucleotides at diagnostic sites that discriminate between retrogene and parental gene were manually confirmed. All oligonucleotide sequences used for PCR and sequencing are available upon request.

### Age of retrocopies

We estimated the age of retroposition events by calculating coding sequence divergence at synonymous sites (*K*_S_) between each retrocopy and the corresponding parental gene. The same analysis was performed for parental genes and their mouse orthologs. Codon sequences were aligned on the basis of the translated sequence alignment using the EMBOSS package [56]. In all alignments, the coding sequence of the parental gene was used as a reference. Pairwise *K*_S_ statistics were estimated using the YN00 program of PAML [57] version 3.14. We note that the ages of retrocopies may be slightly underestimated by this approach, because silent sites are not always completely neutral ([58] and references therein).

Using a phylogenetic dating approach, we determined the age of individual retrocopies by screening for their presence or absence in primate genomes using PCR with primers flanking the insertion site. We confirmed that the insertion site in species not carrying the copy reflects the expected size of the ancestral state (before retrocopy insertion [12]). For five of the retrogenes analyzed in detail, the ancestral state of the insertion site was further confirmed by sequencing. For the two retrogenes *(NACA2* and *PABP3)* present in all anthropoid primates (hominoids, Old World monkey, and New World monkey), we confirmed their absence in lemur and tupaia using several different primer pairs located in their coding regions, as the insertion site could not be amplified using primers in the flanking region.

### Rate of retrogene formation

Pairwise *K*_A_ and *K*_S_ statistics for all retrocopies were estimated using the YN00 program of PAML [57] version 3.14. To estimate *K*_A_/*K*_S_ on the retrocopy lineage itself, we performed the same analysis but compared the retrocopy and the ancestral sequence of the retrocopy at the time point of retroposition (estimated by a maximum likelihood procedure; using the codeml program of PAML [57] and the mouse ortholog of the parent as outgroup). *K*_A_/*K*_S_ is influenced by the GC content at synonymous sites of the parent as well as by the GC content of the genomic region surrounding the retrogene [59]. In particular, retrocopies derived from parental genes with high GC that insert into regions of low GC may show low *K*_A_/*K*_S_ driven by local adaptation to local GC. To test whether GC differences between intact and retropseudogene copies with low *K*_A_/*K*_S_ (<0.5) explain differences in *K*_A_/*K*_S_ between these two types of sequences, we first estimated the GC content at 4-fold degenerate sites and in regions (20 kb) upstream and downstream of the retrocopies, according to the previous analysis [59]. Intact retrocopies and retropseudogenes showed no significant difference when analyzing copies stemming from high-GC (>60% at 4-fold degenerate sites) parents that inserted into low-GC (lower than median value of GC) regions (52 of 130 intact retrocopies versus 60 of 172 retropseudogene copies, *p* = 0.4). Thus, the difference in the distributions for *K*_A_/*K*_S_ < 0.5 between the two types of retrocopies is not accounted for by differences in GC but is likely explained by purifying selection on a number of intact retrocopies.

### Functionality of retrocopies

Codon sequences were aligned on the basis of the translated sequence alignments using the EMBOSS package [56]. Phylogenetic trees were based on the established evolutionary relationships of primates [18]. In the simulation approach used to support functionality of retrocopies, we reconstructed the ancestral state of the retrocopy at the time point of duplication based on this phylogeny using the codeml program of PAML [57] and the parent as an outgroup. Then, we repeatedly simulated the evolution of this ancestral sequence throughout the phylogeny assuming neutral evolution (i.e., point mutations and indels accumulate according to a neutral model of sequence evolution). We used the Kimura-2 parameter model [60] for sequence evolution (assuming a transition/transversion ratio of two), a point mutation rate of 1.0 × 10^−9^ per site per year as suggested previously for hominoids and Old World monkeys [15], and an indel rate of 1.0 × 10^−10^ per site per year [61]. Indels with a multiple of three nucleotides (17%) were assumed to be nondeleterious as they do not disrupt the ORF. The simulations provided a probability (*P*_dis_) for each gene, which corresponds to the number of simulated datasets with a number of deleterious mutations on the different lineages that is smaller or equal to our observation. In parallel, the accumulation of nonsynonymous and synonymous substitutions in the simulated phylogenies was monitored. Thus, we could compare the observed ratio of nonsynonymous to synonymous substitutions to its null distribution estimated by the simulations. The parental genes of the seven retrogenes for which functionality was supported showed low to medium GC content (22%−52%) at 4-fold degenerate, similar to the GC content of the regions flanking their insertions sites (33%−47%). Thus, GC effects (see above; [59]) are unlikely to explain nonsynonymous/synonymous distribution patterns, which are therefore indicative of purifying selection for several cases.

### Selection analysis using PAML

To test for the presence of sites under diversifying selection (*K*_A_/*K*_S_ > 1) on the retrogene lineages, we compared model M1 and model A as implemented in codeml from the PAML package [57] using likelihood ratio tests [62]. Model M1 assumes two classes of sites for the sequences in the whole phylogeny: sites under purifying selection (*K*_A_/*K*_S_ < 1) and neutral sites (*K*_A_/*K*_S_ = 1). Model A adds a third class of sites in the retrogene lineages, with *K*_A_/*K*_S_ as a free parameter, allowing for sites with *K*_A_/*K*_S_ > 1. We also compared this model A to a modified model where *K*_A_/*K*_S_ is fixed at one. Sites under positive selection in the retrogene lineages were identified using the Bayesian approach as implemented in codeml [63]. Note that with respect to *CDC14B2,* the human and chimpanzee sequences have lost the original translation initiation codon (methionine) used by the parental gene (which may have led to the annotation of this gene as a VEGA pseudogene, OTTHUMG00000033880) and gained a putatively new methionine start codon at position 31. The selection tests show similar (statistically significant) results when either the original full-length sequence alignment or a shorter alignment starting from position 31 is used (data not shown).

## Supporting Information

Figure S1 Distribution of *K*_S_ between Parental Genes and Their Orthologs in Mouse (644 KB EPS).

Figure S2 *K*_A_ */K*_S_ on the Retrocopy Lineage: Comparison of the *K*_A_ */K*_S_ Distributions for Intact Retrocopies and Retropseudogenes The mode of the *K*_A_ */K*_S_ distributions is smaller than one (usually expected under neutrality), owing to the effect previously described [59]. White bars correspond to intact retrocopies, and dark bars to retropseudogene copies.
(636 KB EPS).

Figure S3 Simulation Results for *CDC14B2*
See legend of Figure 3.
(7.9 MB PDF).

Figure S4 Simulation Results for *eIF-2-gamma2*
See legend of Figure 3.
(6.6 MB PDF).

Figure S5 Simulation Results for *GMCL2*
See legend of Figure 3.
(6.6 MB PDF).

Figure S6 Simulation Results for *KIF4B*
See legend of Figure 3.
(6.6 MB PDF).

Figure S7 Simulation Results for *NACA2*
See legend of Figure 3.
(6.7 MB PDF).

Figure S8 Simulation Results for *PABP3*
See legend of Figure 3.
(6.6 MB PDF).

Figure S9 Phylogenetic Trees for the Seven Retrogenes Identified in This Study Maximum likelihood *K*_A_ */K*_S_ values and the estimated number of nonsynonymous versus synonymous substitutions (in parentheses) for each branch are indicated.
(791 KB EPS).

Table S1 Dated Retrocopies (107 KB DOC).

### Accession Numbers

The GenBank (http://www.ncbi.nlm.nih.gov/Genbank/) accession numbers for the primate sequences generated for this paper are DQ120612−DQ120720. They are detailed in Table S1. The Ensembl (http://www.ensembl.org/) accession numbers for other genes discussed in this paper are *GMCL1* (ENSG00000087338) and *PABP1* (ENSG00000152520).

## Abbreviations

MYA: million years ago
ORF: open reading frame
