# Supplementary material for: Emergence of Young Human Genes after a Burst of Retroposition in Primates
Source: PLoS Biol. 2005 Oct 11;3(11):e357. doi: 10.1371/journal.pbio.0030357 (PMC1251493; doi:10.1371/journal.pbio.0030357)
Supplement: Supplementary file 2 [file pbio.0030357.sg004.pdf]

A

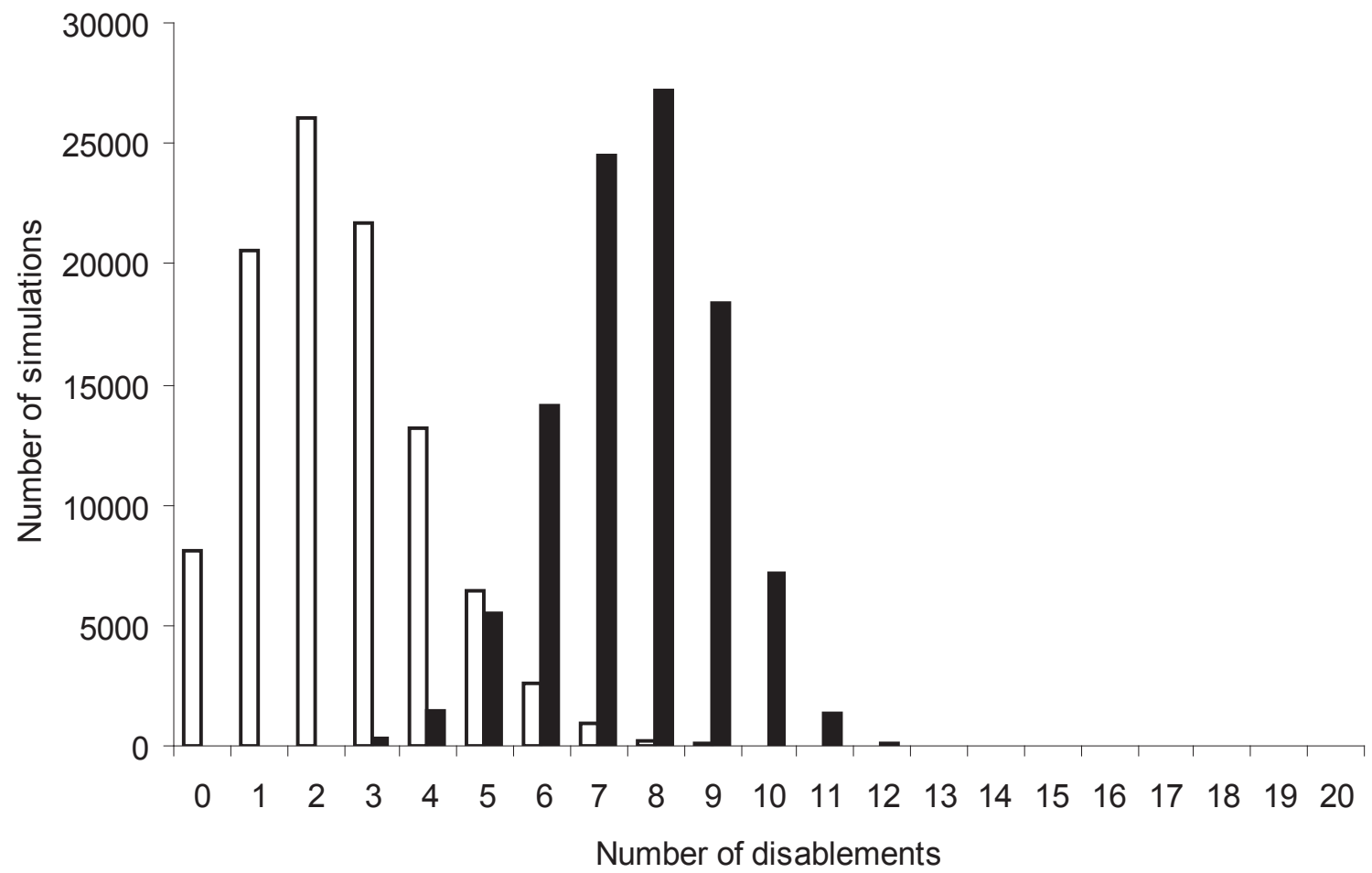

30000
25000
20000
15000
10000
5000
0
Number of simulations
0 1 2 3 4 5 6 7 8 9 10 11 12 13 14 15 16 17 18 19 20
Number of disablements

B

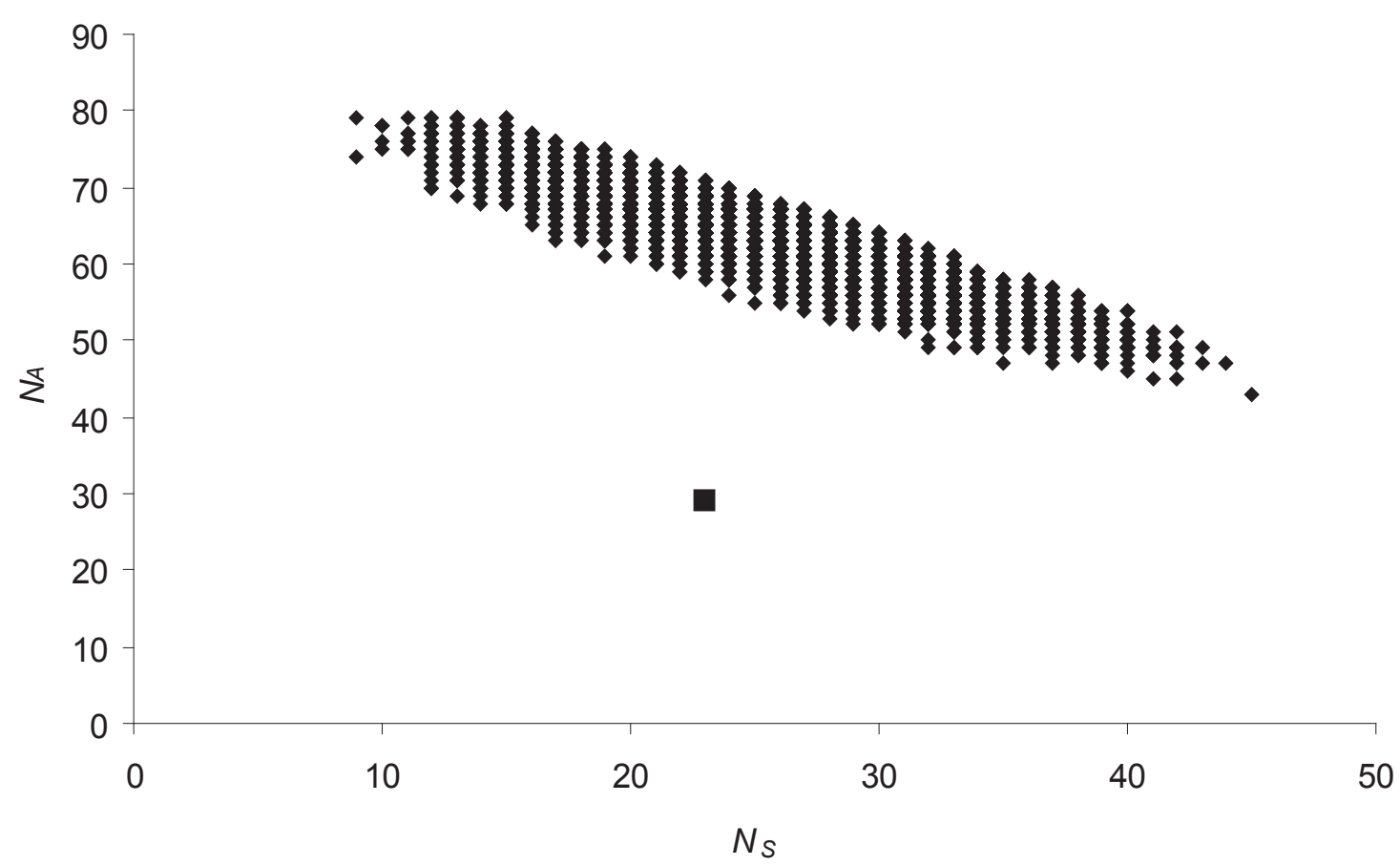

90
80
70
60
50
40
30
20
10
0
N_A
0 10 20 30 40 50
N_S

C

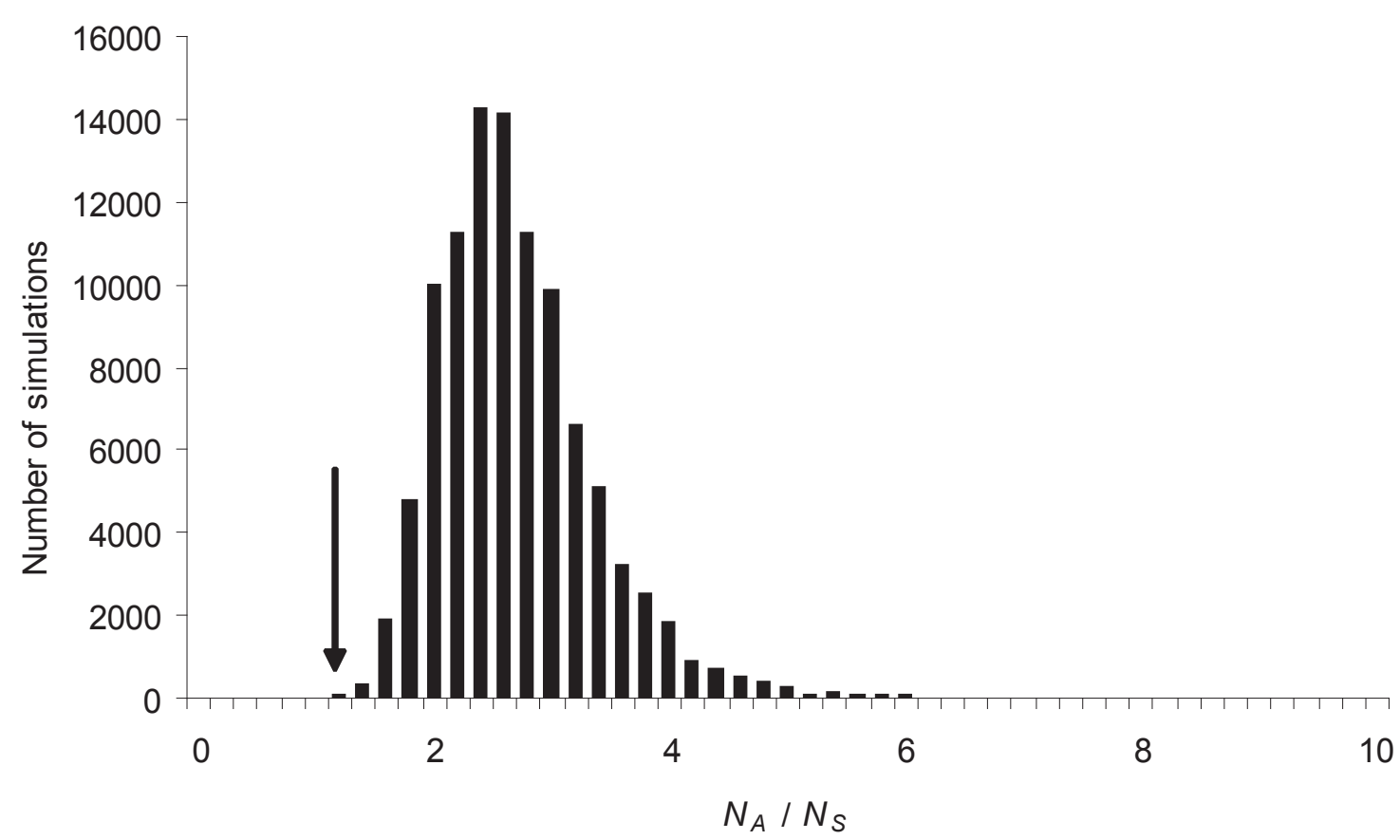

16000
14000
12000
10000
8000
6000
4000
2000
0
Number of simulations
0 2 4 6 8 10
N_A / N_S
