# Supplementary material for: Emergence of Young Human Genes after a Burst of Retroposition in Primates
Source: PLoS Biol. 2005 Oct 11;3(11):e357. doi: 10.1371/journal.pbio.0030357 (PMC1251493; doi:10.1371/journal.pbio.0030357)
Supplement: Supplementary file 4 [file pbio.0030357.sg006.pdf]

A

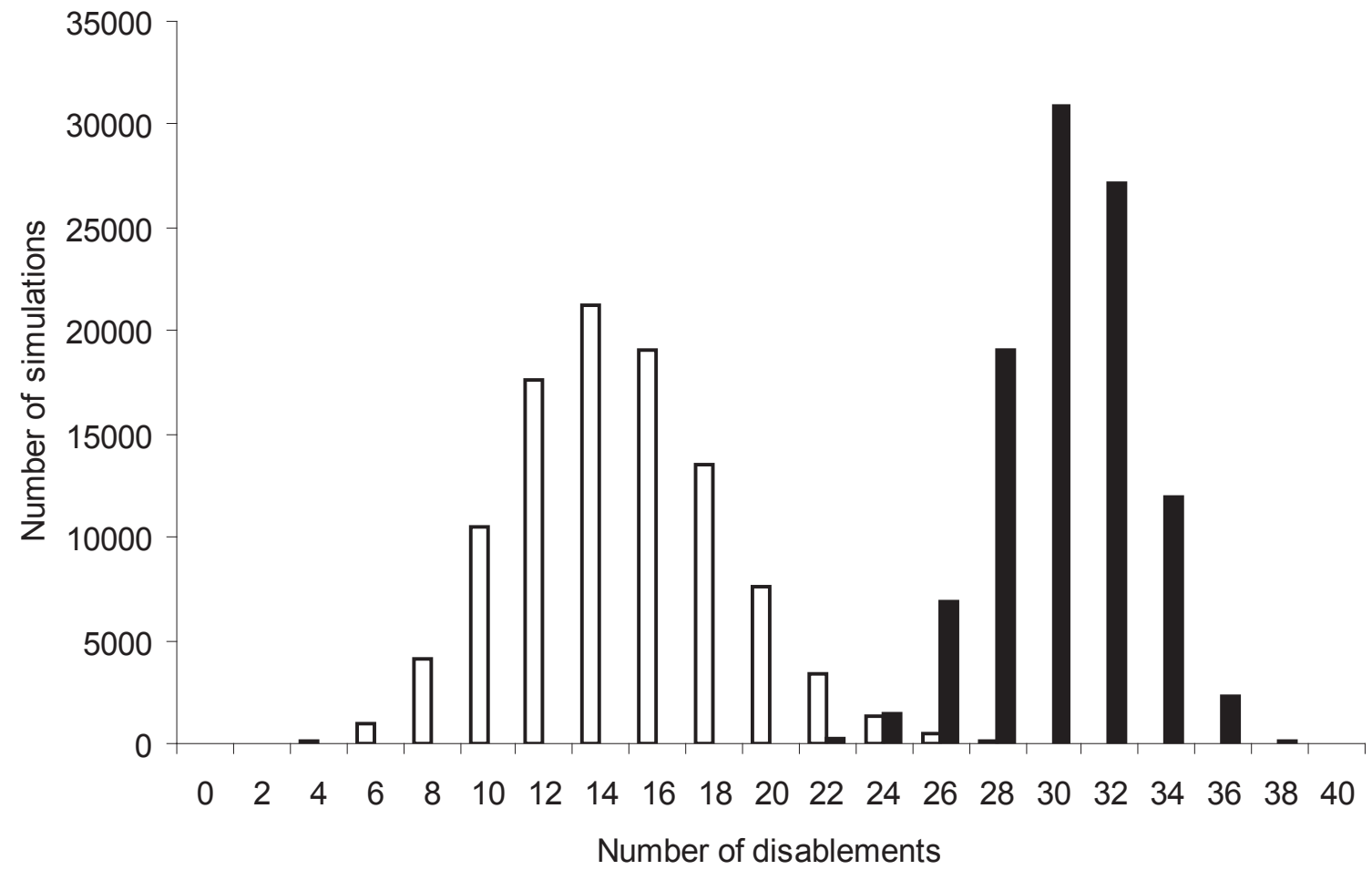

Number of simulations
35000
30000
25000
20000
15000
10000
5000
0
0 2 4 6 8 10 12 14 16 18 20 22 24 26 28 30 32 34 36 38 40
Number of disablements

B

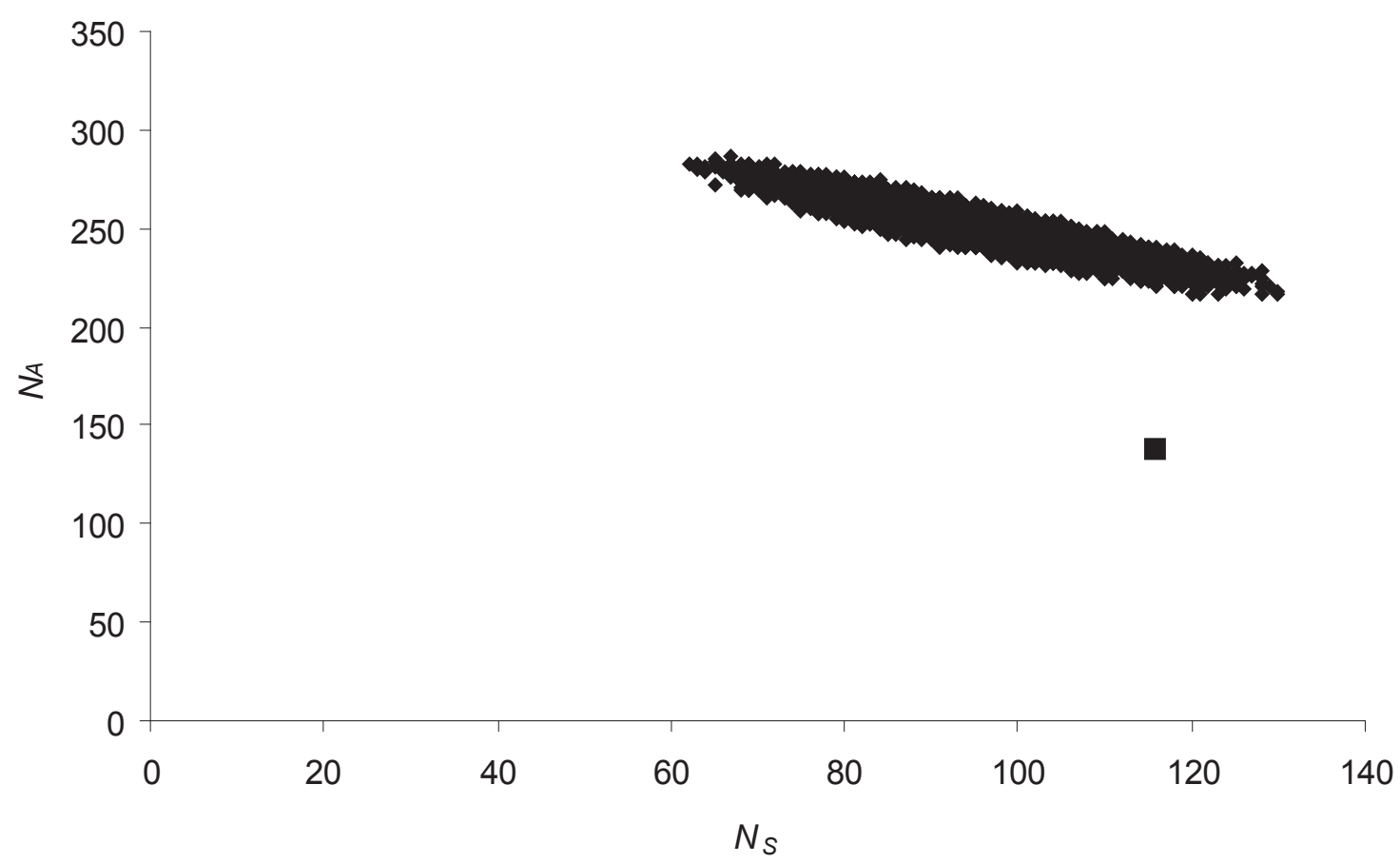

N_A
350
300
250
200
150
100
50
0
0 20 40 60 80 100 120 140
N_S

C

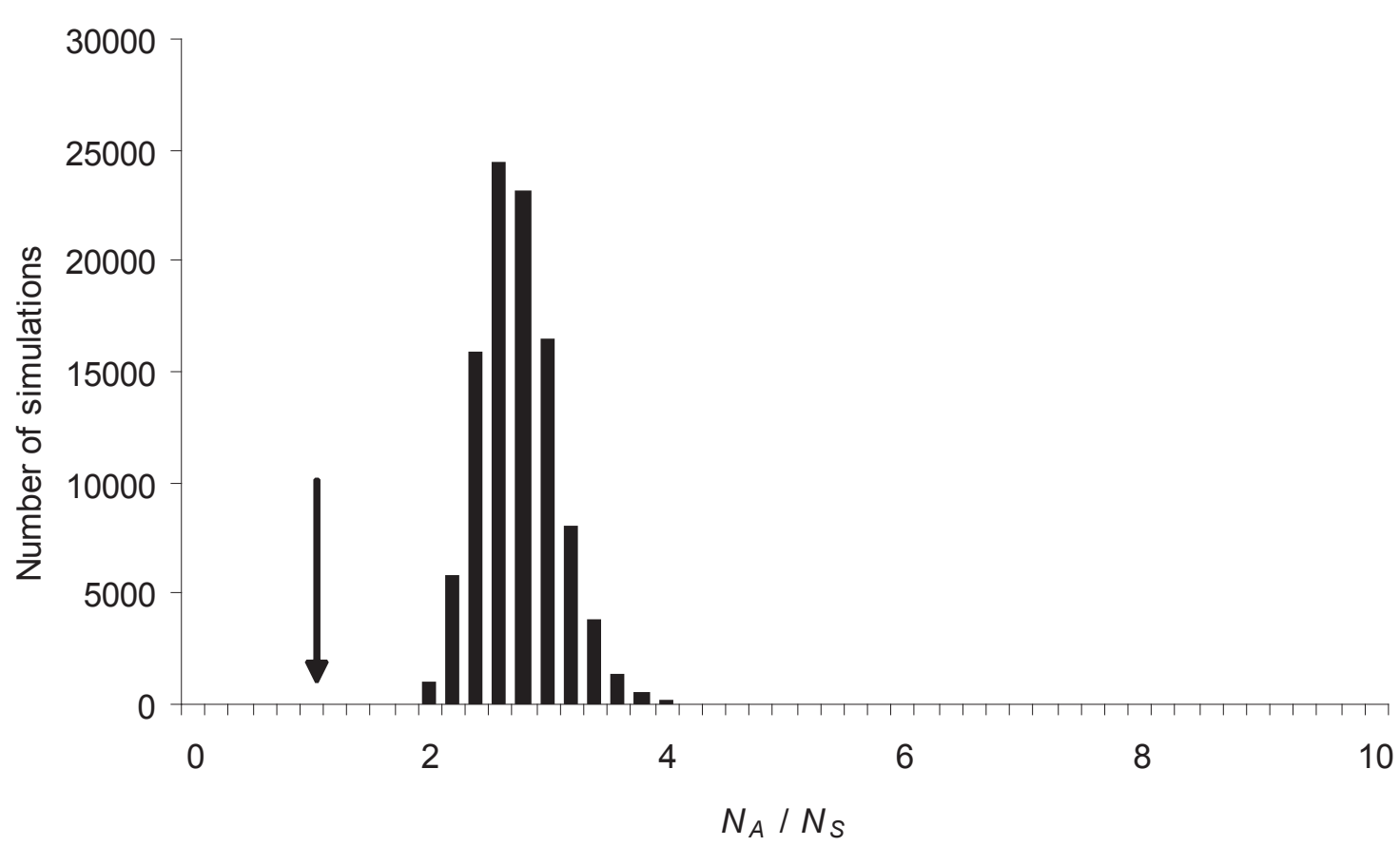

Number of simulations
30000
25000
20000
15000
10000
5000
0
0 2 4 6 8 10
N_A / N_S
