# Supplementary material for: Emergence of Young Human Genes after a Burst of Retroposition in Primates
Source: PLoS Biol. 2005 Oct 11;3(11):e357. doi: 10.1371/journal.pbio.0030357 (PMC1251493; doi:10.1371/journal.pbio.0030357)
Supplement: Supplementary file 6 [file pbio.0030357.sg008.pdf]

A

B

C

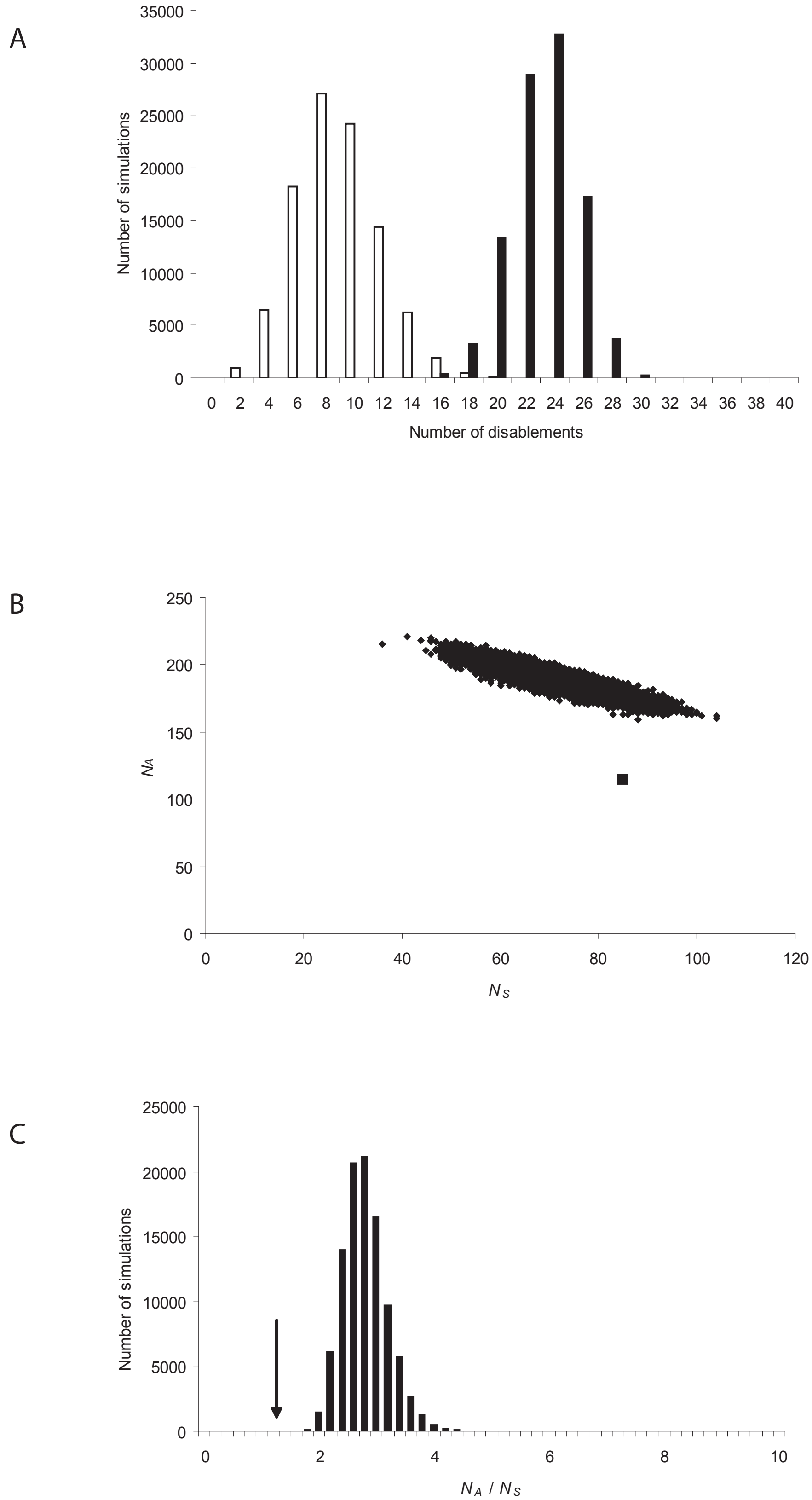

Number of simulations
Number of disablements
N_A
N_S
Number of simulations
N_A / N_S
