# Supplementary material for: Emergence of Young Human Genes after a Burst of Retroposition in Primates
Source: PLoS Biol. 2005 Oct 11;3(11):e357. doi: 10.1371/journal.pbio.0030357 (PMC1251493; doi:10.1371/journal.pbio.0030357)
Supplement: Supplementary file 7 [file pbio.0030357.st001.doc]

Table S1. Dated retrocopies

| Parental gene name ^a^ | Parental location | Ensembl Parental ID | Retrocopy location | Ensembl retrocopy ID | Parental function | *K_S_* ^b^ | CDS length (nt) | Age | GenBank accession number |
| --- | --- | --- | --- | --- | --- | --- | --- | --- | --- |
| *ADH5* | 4 | ENSG00000197894 | 6 | ENSG00000174556 | Alcohol metabolism | 0.054 | 1128 | Hominoids | DQ120656-DQ120660 |
| *C1orf37* | 1 | ENSG00000163444 | 3 | ENSG00000169883 | Unknown function | 0.000 | 1128 | Human |  |
| *C2orf4* | 2 | ENSG00000162959 | 21 | ENSG00000179408 | Unknown function | 0.038 | 885 | African apes | DQ120661-DQ120663 |
| *C6orf68* | 6 | ENSG00000153989 | X | ENSG00000183455 | Unknown function | 0.013 | 879 | Human |  |
| *CDC14B* | 9 | ENSG00000081377 | 7 | ENSG00000178834 | Protein metabolism and modification | 0.135 | 1440 | Hominoids | DQ120635-DQ120639 |
| *CDC20* | 1 | ENSG00000117399 | 9 | ENSG00000186882 | Cell division cycle | 0.051 | 1494 | Hominoids | DQ120664-DQ120668 |
| *CTAGE5* | 14 | ENSG00000150527 | 10 | ENSG00000176822 | Tumor-associated antigen | 0.103 | 2433 | Old World monkeys | DQ120669-DQ120674 |
| *DNAJC9* | 10 | ENSG00000182180 | 20 | ENSG00000125881 | Protein biosynthesis | 0.025 | 756 | Great apes | DQ120675-DQ120678 |
| *eIF-2-gamma* | X | ENSG00000130741 | 12 | ENSG00000180574 | Protein synthesis | 0.046 | 1416 | Hominoids | DQ120619-DQ120623 |
| *FABP5* | 8 | ENSG00000164687 | 13 | ENSG00000118928 | Metabolism of fatty acids | 0.019 | 405 | African apes |  |
| *GMCL1* | 2 | ENSG00000087338 | 5 | ENSG00000185005 | Transcriptional regulation | 0.081 | 1578 | Hominoids | DQ120624-DQ120628 |
| *HMGB1* | 13 | ENSG00000189403 | 3 | ENSG00000132967 | Transcriptional regulation | 0.012 | 633 | Human and Chimpanzee |  |
| *HNRCL* | 14 | ENSG00000092199 | 2 | ENSG00000179412 | RNA processing | 0.011 | 879 | Human |  |
| *KIF4A* | X | ENSG00000090889 | 5 | ENSG00000184732 | Chromosome condensation and segregation | 0.073 | 3657 | Old World monkeys | DQ120629-DQ120634 |
| *NACA* | 12 | ENSG00000196531 | 17 | ENSG00000196861 | Transcriptional regulation and protein biosynthesis | 0.069 | 645 | Anthropoids | DQ120612-DQ120618 |
| *NANOG* | 12 | ENSG00000111704 | 15 | ENSG00000179437 | Cell growth | 0.008 | 915 | Human |  |
| *NIP30* | 16 | ENSG00000172775 | 4 | ENSG00000186384 | Nuclear localisation signal | 0.052 | 762 | Old World monkeys | DQ120679-DQ120684 |
| *NP_006324.1* | 2 | ENSG00000197223 | 10 | ENSG00000181993 | Unknown function | 0.000 | 420 | African apes |  |
| *NP_055642.2* | 1 | ENSG00000058673 | 6 | ENSG00000174560 | Unknown function | 0.022 | 2433 | African apes |  |
| *NSEP1* | 1 | ENSG00000065978 | 14 | ENSG00000197907 | mRNA processing | 0.025 | 894 | African apes | DQ120685-DQ120687 |
| *PABPC1* | 8 | ENSG00000070756 | 13 | ENSG00000151846 | Transcriptional regulation and protein biosynthesis | 0.081 | 1893 | Anthropoids | DQ120640-DQ120646 |
| *PBEF1* | 7 | ENSG00000105835 | 10 | ENSG00000149962 | Cell proliferation | 0.033 | 1419 | Human and Chimpanzee |  |
| *PGAM1* | 10 | ENSG00000171314 | X | ENSG00000186076 | Metabolism of glucids and ethanol | 0.009 | 762 | African apes | DQ120647-DQ120649 |
| *POU5F1* | 6 | ENSG00000137369 | 8 | ENSG00000173317 | Transcription factor | 0.035 | 1077 | Great apes | DQ120688-DQ120691 |
| *PSMB3* | 17 | ENSG00000108294 | 2 | ENSG00000175194 | DNA replication and damage repair | 0.094 | 615 | Great apes | DQ120692-DQ120695 |
| *PTPNS1* | 20 | ENSG00000198053 | 22 | ENSG00000184043 | Cell surface receptor | 0.094 | 1497 | Great apes | DQ120696-DQ120699 |
| *PTTG1* | 5 | ENSG00000164611 | 8 | ENSG00000178717 | Chromosome stability | 0.069 | 606 | Old World monkeys | DQ120700-DQ120705 |
| *RBMX* | X | ENSG00000147274 | 1 | ENSG00000137944 | RNA binding motif protein | 0.050 | 1170 | Old World monkeys | DQ120650-DQ120655 |
| *RHOQ* | 2 | ENSG00000119729 | 14 | ENSG00000183940 | Cell response | 0.018 | 618 | Great apes |  |
| *RPE* | 2 | ENSG00000197713 | 10 | ENSG00000156367 | Metabolism of glucids and ethanol | 0.038 | 684 | Great apes | DQ120706-DQ120710 |
| *SPC2* | 11 | ENSG00000118363 | 1 | ENSG00000189015 | Protein processing | 0.052 | 369 | Human |  |
| *SVH* | 7 | ENSG00000170632 | 3 | ENSG00000178660 | Cell growth | 0.018 | 756 | Human |  |
| *TDGF1* | 3 | ENSG00000163828 | X | ENSG00000183514 | Cell differentiation | 0.006 | 564 | African apes |  |
| *TPI1* | 12 | ENSG00000111669 | 1 | ENSG00000186743 | Metabolism of glucids and ethanol | 0.026 | 747 | African apes | DQ120711-DQ120713 |
| *TPM3* | 1 | ENSG00000143549 | 16 | ENSG00000183022 | Binds to actin filaments | 0.036 | 741 | Great apes | DQ120714-DQ120717 |
| *TXNDC9* | 2 | ENSG00000115514 | 4 | ENSG00000145268 | Cell differentiation | 0.014 | 729 | Great apes |  |
| *UBE2E3* | 2 | ENSG00000170035 | X | ENSG00000181669 | Catalyzes the covalent attachment of ubiquitin to other proteins | 0.013 | 621 | Great apes |  |
| *UBE2S* | 19 | ENSG00000108106 | 17 | ENSG00000175091 | Catalyzes the covalent attachment of ubiquitin to other proteins | 0.026 | 666 | African apes | DQ120718-DQ120720 |

^a^Parental gene names are taken from the HUGO gene nomenclature.

^b^Parent-retrocopy pairwise *K_S_*.
